# PLGA-Based Nanoplatforms in Drug Delivery for Inhibition and Destruction of Microbial Biofilm

**DOI:** 10.3389/fcimb.2022.926363

**Published:** 2022-06-21

**Authors:** Aref Shariati, Zahra Chegini, Ehsanollah Ghaznavi-Rad, Ehsan Nazarzadeh Zare, Seyed Mostafa Hosseini

**Affiliations:** ^1^ Molecular and Medicine Research Center, Khomein University of Medical Sciences, Khomein, Iran; ^2^ Department of Microbiology, School of Medicine, Hamadan University of Medical Sciences, Hamadan, Iran; ^3^ Department of Microbiology, Faculty of Medicine, Arak University of Medical Sciences, Arak, Iran; ^4^ School of Chemistry, Damghan University, 36716-41167, Damghan, Iran

**Keywords:** biofilm, PLGA, poly lactic-*co*-glycolic acid, Pseudomonas aeruginosa, Staphylococcus aeruginosa, Staphylococcus epidermidis

## Abstract

The biofilm community of microorganisms has been identified as the dominant mode of microbial growth in nature and a common characteristic of different microorganisms such as Pseudomonas aeruginosa, Staphylococcus aureus, and Staphylococcus epidermidis. The biofilm structure helps in the protection from environmental threats including host immune system and antimicrobial agents. Thus, the biofilm community has led to a higher prevalence of multidrug-resistant (MDR) strains in recent years. In this regard, the use of a new class of antibiotics, natural compounds, and anti-biofilm enzymes has been considered for the destruction of the microbial biofilm. However, different drawbacks such as low penetration, high susceptibility to degradation, instability, and poor solubility in aqueous solutions limit the use of anti-biofilm agents (ABAs) in a clinical setting. As such, recent studies have been using poly lactic-*co*-glycolic acid (PLGA)-based nanoplatforms (PLGA NPFs) for delivery of ABAs that have reported promising results. These particles, due to proper drug loading and release kinetics, could suppress microbial attachment, colonization, and biofilm formation for a long time. Additionally, PLGA NPFs, because of the high drug-loading efficiencies, hydrophilic surface, negative charge, and electrostatic interaction, lead to effective penetration of antibiotics to the deeper layer of the biofilm, thereby eliminating the microbial biofilm. Thus, PLGA NPFs could be considered as a potential candidate for coating catheters and other medical material surfaces for inhibition and destruction of the microbial biofilm. However, the exact interaction of PLGA NPFs and the microbial biofilm should be evaluated in animal studies. Additionally, a future goal will be to develop PLGA formulations as systems that can be used for the treatment of the MDR microbial biofilm, since the exact interactions of PLGA NPFs and these biofilm structures are not elucidated. In the present review article, we have discussed various aspects of PLGA usage for inhibition and destruction of the microbial biofilm along with different methods and procedures that have been used for improving PLGA NPF efficacy against the microbial biofilm.

## 1 Introduction

Antibiotic resistance and biofilm formation by pathogenic microorganism have become major global health and economic problems for which many researchers worldwide are looking for new and more effective approaches. Biofilms are a community of microbial cells embedded in an extracellular matrix containing exopolysaccharides (EPSs), proteins, DNA, antibiotic-inactivating enzymes, and effluent pumps (Iannitelli et al., 2011b). Many diseases, including chronic rhinosinusitis and endocarditis, are examples associated with the growth of bacterial biofilms (Sanderson et al., 2006; Iannitelli et al., 2011b). Biofilm formation increases the resistance of a microorganism to many antimicrobial agents, with the biofilm-forming bacteria being several times (100–1,000-fold) resistant to antibacterial compounds, immune system, and environmental stresses than their planktonic counterparts. Higher biofilm communities resistant to various antimicrobial agents could be related to their limited penetration to the deeper layers of the biofilm, slowing the growth of bacteria and the molecular interactions between them (Stewart and Costerton, 2001; Kang et al., 2019). Many microorganisms have been shown to have the ability to form biofilms, specifically, *Staphylococcus aureu*s, *Staphylococcus epidermidis*, and *Pseudomonas aeruginosa* are considered bacterial pathogens with the ability to form biofilm communities on various environmental and body surfaces (Deepika et al., 2019; Francisconi et al., 2020).

To combat the bacterial biofilm, various methods such as inhibiting the biofilm formation and improving the penetration of antimicrobial agents into the extracellular matrix have been proposed (Guo et al., 2021). Hence, new anti-biofilm agents including bacteriophages, natural compounds, biomolecules that target the biofilm structure, and various nanoparticles (NPs) have been used to destroy the biofilm and prevent its formation by inhibiting bacterial adhesion (Moghadam et al., 2021).

Studies have shown that the integration of nanotechnology and pharmaceutical sciences greatly helps improve the treatment of biofilm-related infections (Iannitelli et al., 2011b). Drug delivery platforms, photothermal therapy, and photodynamic therapy that are nanotechnology-based strategies have shown great success in the treatment of biofilm infections (Xiu et al., 2021). Various drug delivery systems, such as biodegradable NPs, have proven their superior ability to release drug shipments in a controlled and stable manner (Kamaly et al., 2016). Additionally, nanocarriers have several advantages, including the ability to administer therapeutic compounds directly, the ability to adapt multiple drugs, and the effective delivery of drug-related synergistic effects (Deepika et al., 2019). Furthermore, drug carriers can enhance the stability and release of the compound over time and boost its effectiveness (Wolfmeier et al., 2018).

Biocompatible polymers such as polyethylene glycol (PEG) and poly (lactide co-glycolide) (PLGA) are good carriers in drug delivery. Due to its biocompatibility and biodegradability, PLGA is commonly used in the development of drug delivery platforms (Xiu et al., 2021). In this regard, various drug delivery platforms are made of PLGA. According to the results of various studies, it has been determined that PLGA nanoplatforms (NPFs) can deliver and disperse therapeutic agents in a controlled and stable way (Mundargi et al., 2008; Han et al., 2017). In general, PLGA NPFs have shown sustained release *in vitro* by providing a robust drug delivery system for most encapsulated antibiotics and natural compounds and in many studies have had high biofilm penetration (Han et al., 2017).

Given the importance and applicability of PLGA NPFs for inhibiting and destroying the microbial biofilm in recent studies, in the present review article, we comprehensively investigated the effect of PLGA on the microbial biofilm to promote its wider usage in clinical practice.

## 2 PLGA

NPs are solid particles with a spherical shape ranging in size from 10 to 1,000 nm and are manufactured from natural or synthetic polymers (Ding and Zhu, 2018). A wide range of drugs can be delivered through NPs such as small hydrophilic and hydrophobic drugs, vaccines, and biological macromolecules. These particles also allow targeted administration to specific organs or cells or controlled drug delivery. Recent developments in treatment have increased the demand for a new intelligent drug delivery design with more advanced power, efficiency, and properties to ensure the effectiveness of treatment with sufficient availability and ability of encapsulation (Hosseini et al., 2022).

One of the most important challenges in pharmacology is controlled drug delivery to the human body (Patra et al., 2018). Over recent decades, nanotechnology has arrived in many fields and actuated the whole world to phenomenal developments. Meanwhile, the pharmaceutical industry has not been an exception, and new nanopharmaceutical products have entered different markets (Wang et al., 2021; Mohamed et al., 2022). In the past two decades, the pattern of using nanocarriers in the human body to enhance the efficacy and effectiveness of many drugs, especially anticancer drugs, has been well confirmed in both pharmaceutical and clinical experiments. Nowadays, there are various NP-based systems for targeting drug delivery that is either well-developed or underdeveloped. The primary purpose of employing nanocarriers is to reduce drug breakdown, prevent their side effects, increase drug availability, and drug accumulation at the site of the lesion (Nasrollahzadeh et al., 2019; Said et al., 2022).

**Figure 1:**
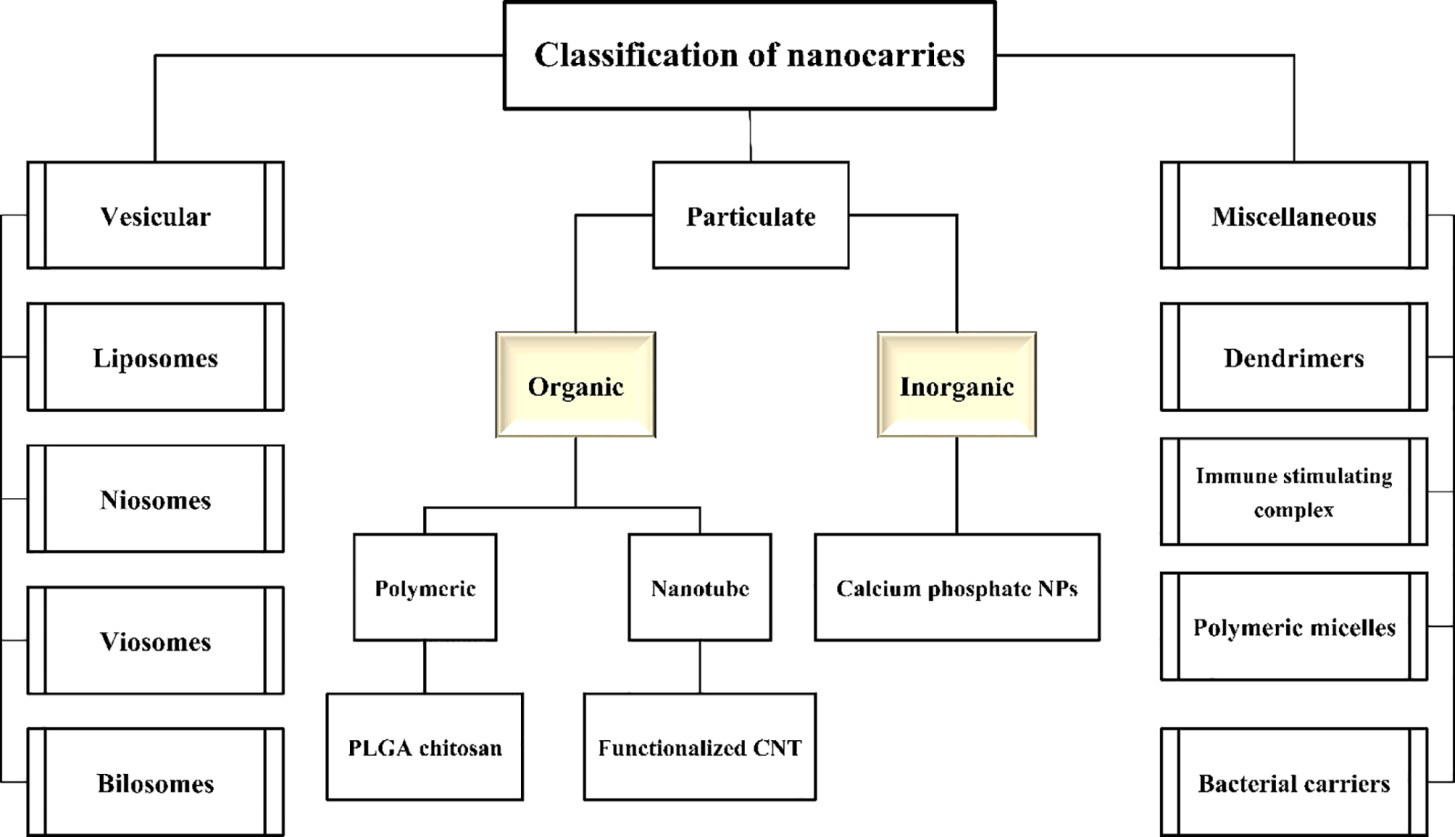
Flowchart of nanocarriers’ classification.

In the common methods of taking drugs, whether oral, injectable, or others, the medication is distributed throughout the body, and the entire body is affected by the effects of the drugs as well as their side effects (Hosseini et al., 2019a). Here, large amounts of medication are required to achieve a specific effect (Singh et al., 2019). So far, many researchers have been addressing this problem, and with the arrival of nanotechnology into the field of pharmaceutical science, the use of this technology in achieving targeted drug delivery has also been considered by many researchers (Hosseini et al., 2019b; Alphandéry, 2020). The use of drug delivery has its advantages over routine medications that can be referred to as follows: preventing the destruction of drugs, increasing the storage time of drugs, storing high amounts of drugs, and having high biocompatibility (Barclay et al., 2019). One of the most important features that makes this technology unique is targeting drug delivery to the specific cell or tissue of the body in cases such as the need to apply low-solubility or insoluble drugs, low-absorption drugs, and gastrointestinal or chemically unstable drugs. Generally, drug nanocarriers can be classified into three main groups: vesicular, particulate, and micelle (Soussan et al., 2009; Banerjee and Pillai, 2019). Each of these groups can be subdivided into smaller subcategories (**Figure 1**).

PLGA is a co-polymer with Food and Drug Administration (FDA) approval (Duskey et al., 2020). These particles are generally synthesized through ring-opening copolymerization of glycolic acid (GA) cyclic dimers and lactic acid (LA) in the presence of catalysts such as tin (II) 2-ethylhexanoate or tin (II) alkoxides, as well as aluminum isopropoxidem. Furthermore, different solvents such as acetone, chlorinated solvents, tetrahydrofuran, and ethyl acetate could be used for PLGA copolymers. To this end, various PLGA copolymers are synthesized using diverse GA : LA ratios where the PLGA crystallinity could range from fully crystalline to fully amorphous depending on the molar ratio and block structure (Gentile et al., 2014; Zare et al., 2020).

Parameters such as the degree of crystallinity, molecular weight porosity of the polymeric network, and LA : GA ratio could affect the biodegradation characteristics of PLGA copolymers. In this regard, the degradation rate rises upon enhancing the GA units in the main chain structure; however, there is an exception, the 50:50 LA : GA ratio, which has the fastest rate of degradation. Furthermore, a higher content of the polyglycolic acid (below 50%) leads to improved degradation (Swider et al., 2018; Zare et al., 2020). Indeed, a higher monomer unit number leads to a shortened degradation time, while a lower glycol content prolongs degradation. Due to the mentioned characteristics, it is possible to design and fabricate PLGA-based NPs with a time-controlled and programmable drug release (Lagreca et al., 2020).

In this regard, fine-tuning the degradation and release rate is gaining increasing attention as researchers keep pushing the boundaries of novel delivery carriers. Notably, different approaches and procedures have been used such as physical blending as well as surface and chemical modification and have been found to be an effective means of release modulation in delivery systems such as oral, parenteral, and tissue- and topical engineering scaffolds (Singh and Singha, 2021).

PLGA is widely employed in the design and formulation of drug delivery systems for biomedical applications due to its biocompatibility, biodegradability, and adaptability in formulation and function. PLGA NPs are used extensively in various applications, including the treatment of neurological/brain disorders and cancer, anti-inflammation, treatment of infectious diseases, and cardiovascular therapy (Won et al., 2021). It can also be encapsulated in a wide range of biologically active molecules including various drugs, proteins, vaccines, and nucleic acids (Ding and Zhu, 2018). Since these nanocarriers can be formulated for systemic injection, oral use, and inhalation, any method of administration can be chosen. While the effectiveness of PLGA NPFs in drug delivery applications has been well confirmed and reported in many research studies, this development makes these micro/nanoformulations undergo clinical trial or currently in use in the pharmaceutical markets, which also has FDA approval (Allavena et al., 2020; Shen et al., 2020; Cunha et al., 2021). Today, of the total number of nano-available pharmaceutical products in the market, PLGA NPFs have received massive consideration due to their strong therapeutic delivery systems, applicability in the treatment of various disorders such as cancer and infectious disease, and commercial impact on micro/nano formed products that have been approved by the FDA. Annual revenues from the sale of these pharmaceutical products are estimated to exceed a total of US $100 billion (Anselmo and Mitragotri, 2014; Zhong et al., 2018; Molavi et al., 2020).

PLGA NPFs can enter cells through endocytosis mediated by clathrin and partly liquid phase pinocytosis. They are rapidly eliminated from the endolysosomes and enter the cytoplasm after 10 min of incubation (Durán-Lobato et al., 2022). In addition to the attractive properties of PLGA NPFs such as small size, high structural integrity, stability, adaptability, and adjustable surface, their physical and chemical properties can be altered. These mentioned properties are obtained from molecular weight, distribution of molecular weight, and composition (GA : LA ratios); as a result, controlling the size, shape, and biodegradability of the prepared nanocarriers is possible, which in turn affects the pharmacokinetics of the encapsulated drug (Rizzo and Kehr, 2021; Rocha et al., 2022). On the other hand, some limitations include their weak drug loading, especially for lipophilic drugs, high explosive release, phagocytic absorption, short half-life, immune response, and uncontrolled distribution, making these drugs not always reliable as a multifaceted and undeniable agent in biomedicine (Kalhapure et al., 2015).

**Figure 2:**
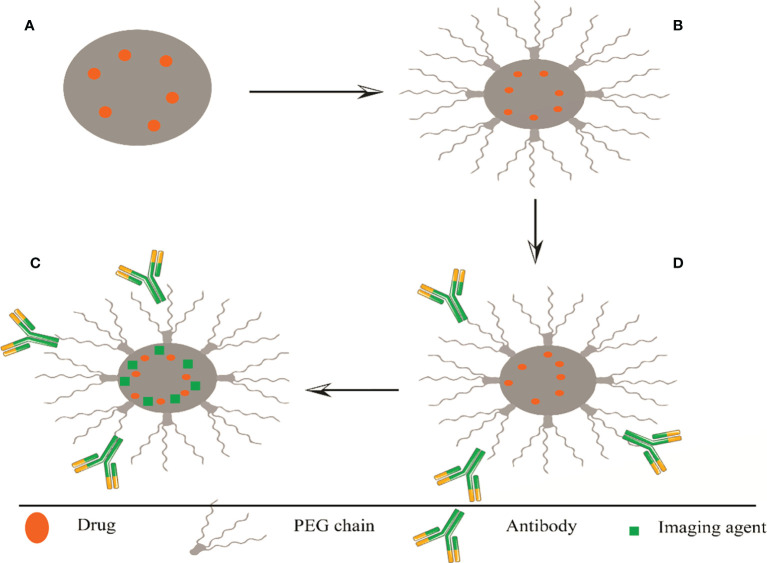
Representation of several methods of surface modifications of PLGA, **(A)** nanosphere and **(B)** PEGylated PLGA. Polyethylene glycol is a non-ionic and hydrophilic polymer; binding to the PLGA surface increases its half-life in the blood circulation. **(C)** Targeted PLGA, increased cell binding and selective internalization through receptor-mediated endocytosis. Target ligands often bind to PEG chains at the nanoparticle surface. The ligands must be optimally conjugated to the nanoparticles to maintain their combined affinity for receptor binding. **(D)** Theragnostics PLGA, load the drug with a fluorescent traceable substance that can detect PLGA. PEG, Polyethylene glycol; PLGA, poly (lactide co-glycolide).

One of the most important biological barriers against controlled drug delivery based on NPs is when the body perceives and recognizes hydrophobic particles as foreign objects. As a result, the reticuloendothelial system (RES) removes these particles from the bloodstream and leads them to the liver or spleen. Additionally, serum opsonin proteins bind to injected NPs and lead them to phagocytosis by attaching to macrophages (Sah et al., 2013; Rocha et al., 2022). To solve this, a hydrophilic layer coats the surface of NPs to cover hydrophobicity. Another advantage of surface modification is to target tumors or organs to enhance selective cell attachment and internalization through receptor-mediated endocytosis. Target ligands are often bonded at the NP surface by bonding to PEG chains; PEG is a hydrophilic and non-ionic polymer that is most common for surface modification. The ligands must be optimally conjugated to the NPs to maintain their tendency to bind to the receptors. Since adequate PEG coating is necessary to prevent detection by RES, ligands must be removed from NP surfaces to avoid protection by PEG chains (Danhier et al., 2012; Ding and Zhu, 2018) (**Figure 2**).

### 2.1 Preparation Methods of Poly Lactic-*Co*-Glycolic Acid

To prepare PLGA NPFs, there are several methods that vary depending on the purpose of the preparation and structure. The drug is either loaded inside the nucleus of a “nanocapsule” or trapped at the surface of the “nanosphere” matrix. In this part, we will review various methods that have been used for the preparation of PLGA NPFs.

#### 2.1.1 Single- or Double-Emulsion Solvent Evaporation Method

**Figure 3:**
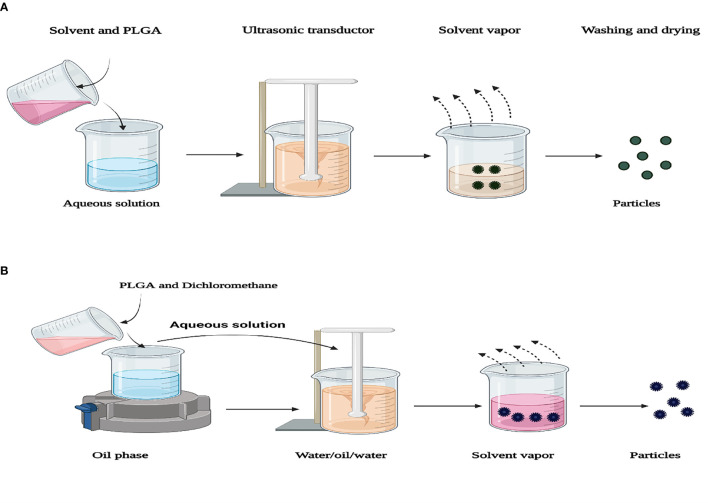
Representation of two preparation techniques of PLGA particles. **(A)** Single-emulsion solvent evaporation. **(B)** Encapsulation of a drug into PLGA nanoparticles using double-emulsion solvent evaporation.

The most prevalent method used to prepare PLGA NPFs is the solvent-emulsion evaporation method that prepares the encapsulation of hydrophobic and hydrophilic drugs. This process involves dissolving a polymer in an organic solvent (such as dichloromethane). The oil emulsion in water (O/W) is prepared by adding water and a surfactant (such as polysorbate-80, poloxamer-188) to the polymer solution. Next, NP droplets are induced by homogenization or sonication. The solvent is then evaporated or extracted, and after centrifugation, the NPs are collected. A modification of this method, W/O/W double-emulsion, was used to enclose hydrophilic drugs, such as peptides, proteins, and nucleic acids. Emulsion evaporation methods are advantageous, as they allow for relative control of particle size and rate of release. The W/O/W method is generally appropriate to encapsulate water-soluble drugs such as peptides, proteins, and vaccines, while the O/W method is suitable for water-insoluble drugs such as steroids (Jain, 2000; Luo et al., 2009). Nevertheless, these mentioned methods are also associated with inherent flaws and problems, especially bio-macromolecule instability. NP size can be controlled by adjusting the stirring speed, type, and amount of dispersing agent, organic and aqueous phase viscosity, and temperature (Zhang et al., 2021) (**Figure 3**).

#### 2.1.2 Spray-Drying Method

**Figure 4:**
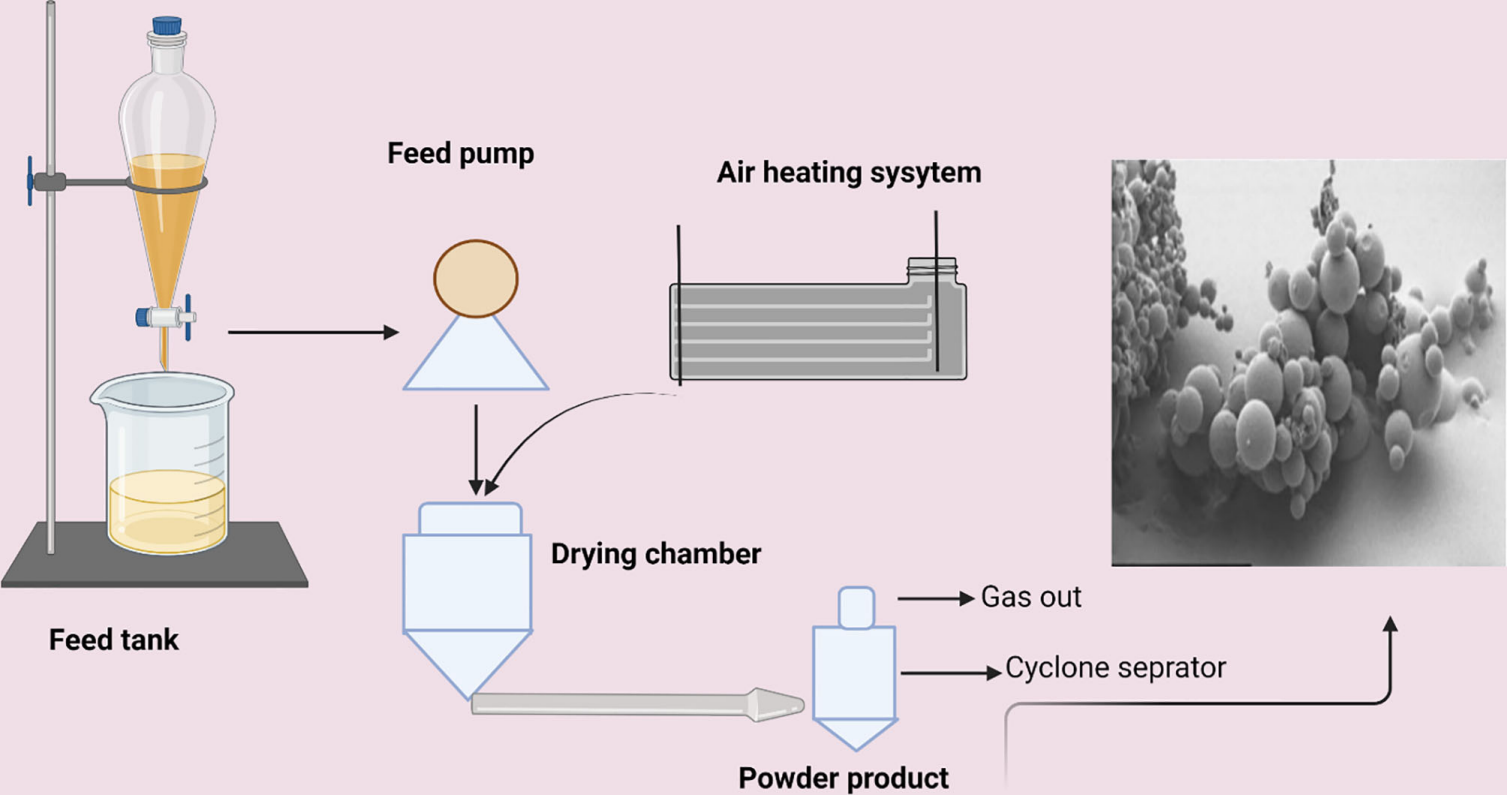
Representation of the spray-drying process.

Spray-drying is a quick and comfortable method to prepare PLGA in smooth conditions with very slow processing parameters, thus making it usable in industrial scaling. In this process, drug-loaded microspheres are sprayed with a solid dispersion in oil or an emulsion of water in oil in a stream of heated air. The type of drug (hydrophilic or hydrophobic) determines the appropriate solvent for the process. The nature of the solvent used, the solvent evaporation temperature, and the feed rate affect the morphology of the final product. This is the most common method to encapsulate drugs and proteins in microparticles without significant loss of biological activity. However, two main drawbacks of this method are microparticle attachments to interior walls of the spray-dryer and difficulty to control particle size, while the yield is average for small batches (Guo et al., 2014; Wan et al., 2014). There are different spray-drying techniques to prepare micro-PLGA particles for proteins, peptides, and DNA delivery (Takada et al., 1995) (**Figure 4**).

#### 2.1.3 Nano-Precipitation Method

It is a single-phase process known as the solvent transfer method and performed using systems containing three basic components: polymer, polymer-solvent, and non-solvent polymer. This technique is usually used to trap hydrophobic and hydrophobic drugs but is also applicable for hydrophilic drugs. Polymer and drugs dissolve in a polar solvent and are water-miscible such as acetone, acetonitrile, ethanol, or methanol. The solution is poured in a controlled manner (add dropwise) in an aqueous solution with surfactant. NPs are formed immediately with the rapid release of the solvent. Finally, the solvent is removed under reduced pressure (Takada et al., 1995; Astete and Sabliov, 2006). There are also other techniques to synthesize PLGA including the emulsification reverse salting-out method (Naskar et al., 2021), phase separation (Mckenna et al., 2019), simple mechanical stirring (Operti et al., 2021), high shear mixing (HSM), and high-pressure homogenization (HPH) (Operti et al., 2018).

## 3 Pseudomonas

*P. aeruginosa* is known as one of the most important opportunistic bacterial pathogens found in many environments and leads to a high rate of deadly hospital-acquired infections (Bahramian et al., 2019). The results of recent investigations have shown a high mortality rate for *P. aeruginosa*-associated infections mainly in patients with predisposing conditions such as cancer, cystic fibrosis, urinary tract infection, and burn wound infections (Bahramian et al., 2019; Glen and Lamont, 2021). Different adhesion factors such as flagella, type IV pili, and biofilms cause adhesion and survivor of this bacterium on various surfaces, medical devices, and water (Remold et al., 2011).

It is now almost accepted that most bacterial species could form a biofilm in a cyclic process, although the first biofilm developmental life cycle has been reported for *P. aeruginosa* (Park and Sauer, 2021). The biofilm community of this bacterium could lead to chronic infections such as chronic pneumonia in patients with cystic fibrosis because of the higher resistance to environmental challenges such as diverse disinfectants, irradiation treatments, antibacterial agents, and phagocytosis by neutrophils. Additionally, biofilms can contain dormant antibiotic-insensitive persister cells (Glen and Lamont, 2021; Reynolds and Kollef, 2021).

Recent studies have reported that a higher dosage of antibiotics is needed for the destruction of the *P. aeruginosa* biofilm community compared to the planktonic cells due to the limited antibiotic penetration into the complex polysaccharide matrix (glycocalyx) of biofilms (Spoering and Lewis, 2001; Ma et al., 2009). Thus, the biofilm is implicated in the increasing prevalence of multidrug resistant (MDR) *P. aeruginosa* strains in recent years, with scientists searching hard for new anti-biofilm agents to control it. In this regard, the delivery of various antibacterial agents such as antibiotics, natural compounds, and enzymes by PLGA platform has also been considered for eliminating *P. aeruginosa* biofilm, and in this section, we will review these studies.

Hua et al., 2014 successfully encapsulated ciprofloxacin (CIP) in PLGA magnetic micro/nanoparticles using W/O/W method. Scanning electron microscopy (SEM) revealed spherical shapes and narrow size distributions for PLGA magnetic particle-encapsulated CIP. The total drug release was 95% and 80.3% from NPs and microparticles, respectively. Additionally, an external oscillating magnetic field triggered the release of CIP from PLGA magnetic particles that then continuously released for 2 weeks after triggered release. Notably, the sustained release of CIP from the PLGA microparticles reduced 20.4% of bacterial activity in the P. aeruginosa biofilm, while this rate was 25.8% for PLGA NPs. Thus, the encapsulation of CIP in PLGA NPs could lead to better antibacterial activity in comparison to the PLGA microparticles. Altogether, CIP maintained its antimicrobial activity after encapsulation and triggered release; thus, the authors suggested that the application of PLGA magnetic particles should be considered as facilitators of drug delivery and drug release switches in future studies (Hua et al., 2014).

In another investigation, NPs composed of a PLGA core and D-α-tocopheryl polyethylene glycol 1000 succinate (TPGS) were again used for the site-specific delivery of azithromycin to the P. aeruginosa biofilms via aerosol direction. The synthesized NPs were self-assembled via a nanoprecipitation process using a facile microfluidic method. The enzymatically cleavable shell of TPGS led to the acceptable penetration of TPGS-PLGA NPs to the mucus and deeper layers of the biofilm, thereby boosting the azithromycin killing efficacy against the biofilm community of P. aeruginosa. Notably, confocal laser scanning microscopy (CLSM) showed that PLGA NPs were fundamentally localized at the superficial layer of the biofilm, while TPGS-PLGA hybrid NPs penetrated to the deeper layer of the biofilm. Thus, it seemed that the hydrophilic moiety of TPGS successfully reduced the binding and interaction of the biofilm EPS and NPs, thereby causing enough penetration of the drug to the biofilm. In this regard, quantitative analysis revealed that azithromycin-loaded TPGS-PLGA hybrid NPs presented approximately 2.5 times greater bactericidal effects in comparison to the azithromycin alone at 6 h. These data suggested that the synthesized NPs could boost the azithromycin site-specific delivery and enhance the drug concentration in the P. aeruginosa biofilm. Hence, the authors introduced the TPGS-PLGA hybrid NPs as a suitable drug delivery system for releasing antibiotics in a sustained manner and for overcoming the various barriers in the treatment of microbial biofilm-associated infection (Wan et al., 2019).

These data support the findings by Sylvia et al., who used TPGS-PLGA hybrid NPs and octenyl succinic anhydride-modified low-molecular weight hyaluronic acid (OSA-HA) nanogels to enhance the efficacy of azithromycin for destroying the P. aeruginosa biofilm. The results indicated that both delivery systems could penetrate the bacterial biofilm and enhance the antivirulence and antibacterial activity of azithromycin. Furthermore, TPGS-PLGA hybrid NPs and OSA-HA nanogels prevented biofilm formation of P. aeruginosa and removed the established biofilm of this bacterium at considerably lower doses of azithromycin in comparison to the non-formulated form of this antibiotic. Note that smaller TPGS-PLGA hybrid NPs revealed longer retention in the biofilm in comparison to the hydrophilic OSA-HA nanogels (Kłodzińska et al., 2019).

In 2019, in another study, the authors used PLGA-methyl ether-block-PEG micelle to boost the performance of piperacillin/tazobactam against the biofilm of clinical isolates of P. aeruginosa with resistance to various antibiotics. Different analyses showed a semi-spherical morphology, a surface charge of 2.98 mV, and a mean diameter of less than 30 nm for piperacillin/tazobactam PLGA-PEG micelle. As compared to the free drug form, this micelle suppressed bacterial movement more effectively, eradicated biofilm communities, and lowered the minimum inhibitory concentration (MIC) (Morteza et al., 2019). In this regard, another examination for degrading the *P. aeruginosa* biofilm also encapsulated tobramycin on the PLGA-PEG with negative zeta potentials, spherical shape, and particle sizes of 896–902 nm (microparticles) and 225–231 nm (NPs). These NPs significantly enhanced the anti-biofilm activity under fluidic and static experimental conditions in artificial mucus. Interestingly, 0.77 mg/l of encapsulated tobramycin killed bacteria in the biofilm community, while 1,000 mg/l of mixtures of particles and tobramycin or free tobramycin were not capable enough to suppress the *P. aeruginosa* biofilm. Notably, tobramycin-PEG-PLGA NPs did not show a cytotoxic effect against human lung epithelial cells. Thus, this drug delivery platform could boost the efficacy of tobramycin against the bacterial biofilm, even aminoglycoside-resistant isolates (Ernst et al., 2018).

Amikacin and gentamycin were other members of the aminoglycoside family that have been loaded in PLGA NPs to eliminate the *P. aeruginosa* biofilm. Amikacin-PLGA NPs, with a zeta potential of 29.8 ± 1.5 mV and a mean diameter of 447 ± 7 nm, were distributed uniformly all along the biofilm thickness and penetrated through the entire biofilm thickness of *P. aeruginosa*. Additionally, CLSM analysis indicated that these NPs dramatically reduced the bacterial biofilm when compared to the free medication. The higher penetration of the amikacin-PLGA NPs than the free drug could be related to the NP negative charge and the resulting electrostatic interaction (Sabaeifard et al., 2017).

Additionally, researchers claimed that their synthesized PLGA particle-encapsulated gentamicin demonstrated regulated gentamicin release for up to 16 days. These particles significantly enhanced the anti-biofilm effect of gentamycin against *P. aeruginosa*. Notably, in this study, the authors used the 96-h peritoneal murine infection model for the *in vivo* assessment of synthesized particles. Compared to the free gentamycin, PLGA particle-encapsulated gentamicin effectively reduced bacterial count (from peritoneal lavage) and inflammatory cytokines. In this regard, the authors suggested that the controlled release of gentamicin NPs could have enhanced the efficacy over free gentamicin (Abdelghany et al., 2012).

Finally, Huang et al. (2020) reported that combining carbon quantum dots (CQDs) with the conventional PLGA NPs could reduce some drawbacks of these NPs such as premature burst release and incapability of enough drug loading as well as their on-demand release at the site of action. Hence, the authors used the microfluidic method and incorporated CQDs into the PLGA NPs (CQD-PLGA hybrid NPs). These NPs showed proper loading capability of tobramycin and azithromycin, stimulus-responsive release of the drugs upon laser irradiation, and good biocompatibility with the eukaryotic cells. Additionally, azithromycin-loaded CQD-PLGA hybrid NPs killed more bacterial cells in the *P. aeruginosa* biofilm community in comparison to the free azithromycin. This increased killing efficacy could be related to the delivery of antibiotics in the specific site of action by NPs, thereby elevating the drug concentration in deeper layers of the biofilm. These results suggested that CQD-PLGA hybrid NPs could be a promising approach for the inhibition of the bacterial biofilm due to the high antibiotic loading capacity and reduced premature burst release (Huang et al., 2020).

The use of the various natural compounds for inhibition of the microbial biofilm has been considered in recent years, and some promising results have also been reported. Nevertheless, poor solubility in aqueous solutions, instability, and volatility are some disadvantages of natural compounds limiting their major use in clinical settings. In this regard, researchers are interested in loading natural compounds on various drug delivery platforms such as PLGA. For example, Kim et al. (2015) reported that incorporation of PLGA with *Cinnamomum* bark oil and its main constituent, cinnamaldehyde, effectively inhibited the biofilm formation of *P. aeruginosa* and *Escherichia coli.* As a result, the scientists recommended that polymer coatings containing natural chemicals might be utilized to limit the formation of the bacterial biofilms on various surfaces such as food processing and biomedical surfaces (Kim et al., 2015). In a recently published study, authors used the emulsification solvent diffusion method and encapsulated Hydroethanolic Extract of Red Propolis (HERP) in PLGA for the destruction of the *P. aeruginosa* biofilm. These NPs were spherical with a size range of 42.4–69.2 nm, with encapsulation efficiencies of 96.99%. HERP-PLGA NPs showed a promising inhibitory effect against the bacterial biofilm. The authors suggested that the penetration of hydrophobic HERP through bacterial cells increased not only due to the small size of PLGA NPs but also because of their hydrophilic surface. The continuous release of HERP from PLGA NPs makes the HERP-PLGA NP a suitable alternative for local antibiotic therapy to inhibit the bacterial biofilm that reduces systemic side effects and patient complications (De Mélo Silva et al., 2020).

In line with the mentioned studies, another investigation also functionalized PLGA and dextran micelles with curcumin, one of the main phytochemical ingredients of *Curcuma longa*. The findings indicated that the synthesized NPs (hydrodynamic diameters 498.7 ± 35.4 nm) have a good penetration to the *Pseudomonas* species biofilm, leading to enhanced antibacterial activity of the conjugated micelles when compared to free curcumin. Additionally, curcumin micelles showed an antibacterial function toward biofilm-embedded cells and effectively disrupted the preformed biofilm. Thus, curcumin conjugation on the micelle structure could enhance curcumin solubility and its transport in the bacterial suspension, which in turn accentuated its antimicrobial capacity. Furthermore, the high biocompatibility of polysaccharides present within the biofilm EPS with dextran presence in the NPs could lead to greater incorporation of micelles into the biofilm architecture (Barros et al., 2021).

In a recently published study, the authors considered that by applying pyruvate dehydrogenase and eliminating pyruvate supply in the biofilm community, it is possible to trigger biofilm bacteria to disperse from the surface-associated mode of growth into the surrounding environment. Thus, it is conceivable that using pyruvate dehydrogenase, it is possible to revert biofilm bacteria to the planktonic mode of growth. However, in clinical settings, direct use of enzymes is not practical, as enzymes are susceptible to denaturation under different storage conditions. In this regard, the authors immobilized pyruvate dehydrogenase in PLGA and prepared NPs with an average size of 266.7 ± 1.8 nm. After 6 days of incubation at 37°C, these NPs remained active and successfully destroyed the *P. aeruginosa* mature biofilms by depleting pyruvate supply. These data suggested that pyruvate dehydrogenase-PLGA NPs lead to biofilm dispersion and could render bacteria susceptible to common antibiotics; accordingly, these NPs should be considered for the treatment of *P. aeruginosa* biofilm-associated infections (Han et al., 2019). Baelo et al. (2015) also functionalized CIP-loaded PLGA NPs with DNase I using a green solvent-based method. The authors assumed that the synthesized NPs can disrupt the bacterial biofilm in two distinct ways: releasing CIP in a controlled fashion and degrading the extracellular DNA that stabilizes the biofilm matrix. The results were in line with their theory as DNase I-PLGA NPs not only reduced 95% of the *P. aeruginosa* biofilm production but also eradicated more than 99.8% of the mature biofilm. The described anti-biofilm effect, on the other hand, was not seen for free drug or a combination of DNase I and free drug. Additionally, the synthesized NPs showed low cytotoxicity against 774 macrophages (Baelo et al., 2015).

As such, as mentioned in the above sections, different characteristics such as proper size, drug loading, drug release kinetics, and low cytotoxicity against eukaryotic cells make PLGA NPFs an applicable candidate for the destruction of *P. aeruginosa* biofilm-associated infections such as cystic fibrosis. This is because encapsulation of various anti-biofilm agents with PLGA could enhance inhaled cystic fibrosis therapy. However, the effectiveness of various PLGA formulations should be evaluated in inhaled antimicrobial therapy in *in vivo* models to completely explore the potential of these platforms.

## 4 Staphylococcus aureus

MDR *S. aureus*, especially methicillin- and vancomycin-resistant strains, has led to huge financial and social burdens in clinical settings that have been increasing in recent years (Wu et al., 2021). In addition to the frequent occurrence of antimicrobial-resistant strains, *S. aureus* often resides within biofilms at the site of infection (Van Den Driessche et al., 2017). The ability of this bacterium to form a biofilm is the main virulence factor related to chronic infections such as the chronicity of wounds (Simonetti et al., 2021).

Additionally, the formation of biofilm by *S. aureus* on different medical surfaces, such as catheters, is an important problem in healthcare-associated infections (Ceylan and Ugur, 2015). Accordingly, researchers are trying to enhance the efficacy of antibiotics against the *S. aureus* biofilm; meanwhile, other possibilities to the existing antibiotics against *S. aureus* biofilm-associated infections are still of interest (Taubes, 2008). In this regard, recent studies have used PLGA NPFs for this purpose.

Ficai et al. (2018) hypothesized that magnetite NPs could enhance the therapeutic efficacy of single antibiotics (even at low drug concentrations) due to the specific nanosize-related properties. Additionally, these NPs could be considered active carriers of the antibiotic, as their eventual exposure to external magnetic fields could lead to the complete release of antibiotics and enhance their efficacy. Furthermore, the mentioned effects could be boosted by applying PLGA matrix, since by increasing local temperature due to the infection and inflammatory processes, the PLGA matrix will enable the local release of antibiotic-coated NPs, thus providing enhanced antibacterial activity (Grumezescu et al., 2014a; Ficai et al., 2018).

In this regard, the authors firstly functionalized iron oxide particles with cefepime (Fe3O4/CEF), and afterward, using the matrix-assisted pulsed laser evaporation method, they coated these particles with PLGA (PLGA-Fe3O4/CEF). Covering the glass slides with PLGA-Fe3O4/CEF significantly suppressed *S. aureus* biofilm formation for at least 72 h. Thus, this nanosystem releases the antibiotic continuously and in a controlled manner and enhances the potential of the antibiotic for the destruction of the bacterial biofilm. Accordingly, PLGA-Fe3O4/CEF could be considered a promising anti-biofilm coating for implantable devices (Ficai et al., 2018).

Note that various etiological factors such as bone necrosis and compressed vascular channels limit antibiotic penetration to the active site of infection, thus making osteomyelitis a persistent infection. As such, local antibiotic therapy could be a better therapeutic approach in comparison to the systemic administration of antibiotics. In this regard, recent studies due to the various features such as sustained drug release capabilities and acceptable biocompatibility have used PLGA-based drug delivery platforms for local delivery of antibiotics for the destruction of the bacterial biofilm and treatment of osteomyelitis (Bastari et al., 2014).

In one of these studies, mesoporous bioactive glass (MBG) was loaded with vancomycin, after which freeze-drying fabrication was used to prepare a bone tissue-engineering scaffold combining PLGA and MBG/vancomycin. Different analyses revealed that this compound could provide sustained release of antibiotics lasting for more than 8 weeks. In addition, coating the scaffold with vancomycin promoted antibacterial and anti-biofilm activity against *S. aureus* without detrimental activity on cytocompatibility. Indeed, the PLGA scaffold with vancomycin-loaded MBG yielded an initial fast release of drug (beneficial for the acute phase of infections) followed by a relatively slow release, which successfully suppressed the adhesion and biofilm formation of *S. aureus*. Hence, the authors suggested this scaffold as a suitable candidate for the treatment of *S. aureus*-associated osteomyelitis due to its anti-biofilm properties (Cheng et al., 2018).

In another investigation, W/O/W double-emulsion solvent evaporation technique was again used for preparing moxifloxacin-PLGA and rifampicin/moxifloxacin-PLGA microspheres for local delivery treatment of *S. aureus*-associated osteomyelitis. Notably, both of these antibiotics were used for the treatment of osteomyelitis. The results indicated that compared to the moxifloxacin-PLGA microsphere, rifampicin/moxifloxacin-PLGA microspheres showed better loading rate, stability, and slow drug release as well as anti-biofilm effects. Collectively, rifampicin/moxifloxacin-PLGA microspheres have a potential for the treatment of osteomyelitis because of their different characteristics such as local drug delivery, suppression of biofilm formation, and biodegradability (Qiao et al., 2019).

In another drug delivery platform for inhibition of *S. aureus* biofilm-associated osteomyelitis, Bastari et al. (2014) loaded levofloxacin into PLGA particles coated with calcium phosphate (CaP). The CaP coating lowered burst release, however, providing continuous release of the antibiotics for up to 4 weeks. Furthermore, the synthesized particles suppressed *S. aureus* biofilm formation and disrupted the established biofilm community of this bacterium. Thus, inhibition of the biofilm formation and destruction of the mature biofilm by levofloxacin-PLGA/CaP particles make these particles a novel therapeutic agent for the prevention of osteomyelitis recurrence after surgery and treatment of this infection mainly when it has reached the stage of attachment and maturation of the biofilm on the bone surface (Bastari et al., 2014).

In addition, Ashbaugh et al. (2016) used the mouse model of methicillin-resistant *S. aureus* (MRSA) biofilm-associated orthopedic implant infection for evaluating PLGA-based drug delivery platform efficacy in the treatment of osteomyelitis. To this end, PLGA nanofibers embedded in a poly (Ɛ-caprolactone) (PCL) film were developed to locally codeliver vancomycin/rifampicin (VAN/RIF) from the implant surface. Based on the animal model results, antibiotic-loaded coatings effectively prevented bone/joint tissue infection and MRSA biofilm formation on the implant; additionally, they were biocompatible with enhanced osseointegration. These data suggested that these antibiotic-loaded coatings should be considered as an applicable tailor for the delivery of conventional antibiotics from different prostheses or metallic implantable devices to effectively reduce biofilm-associated osteomyelitis in patients (Ashbaugh et al., 2016).

**Figure 5:**
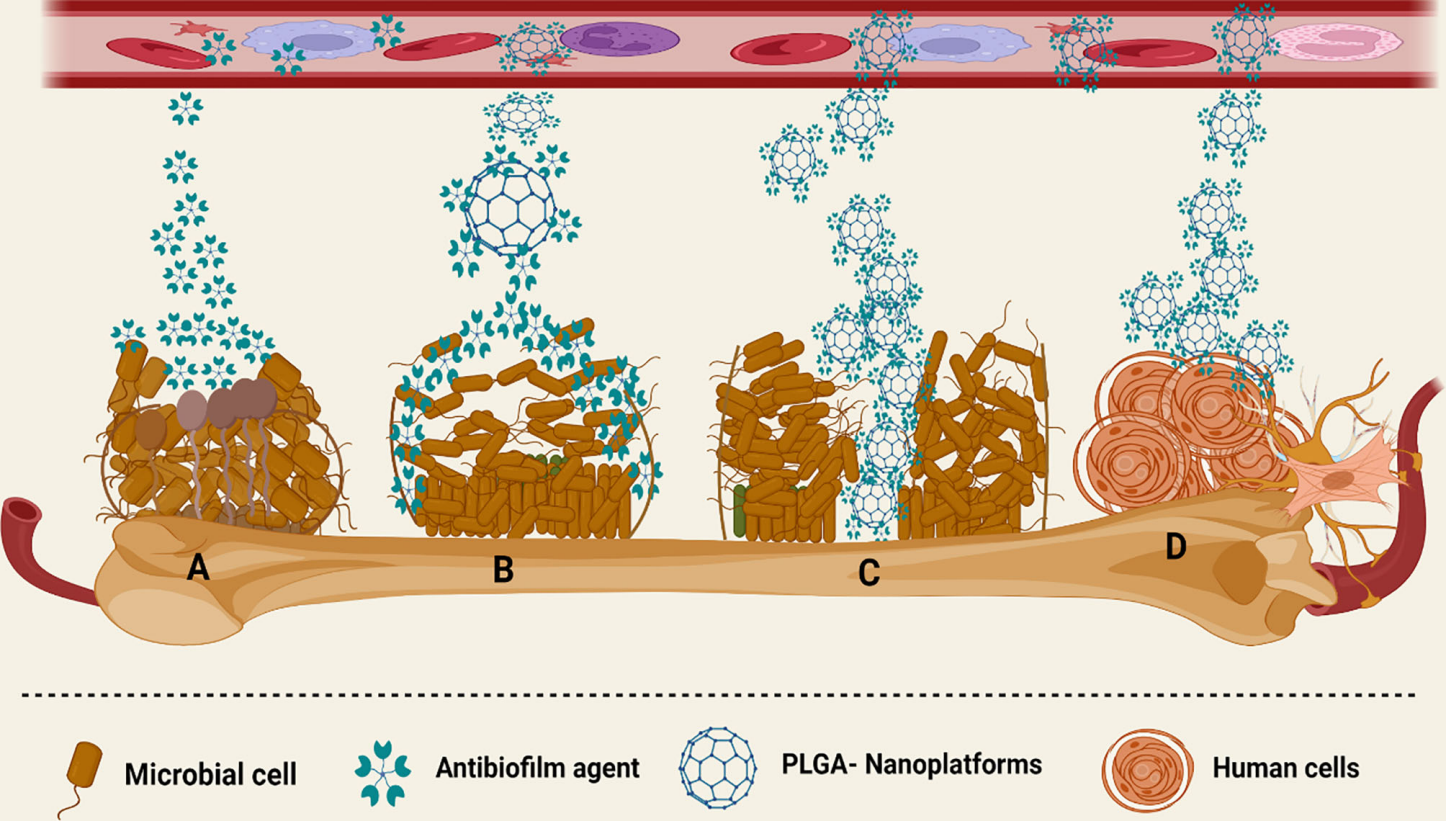
Different interactions of PLGA-based nanoplatforms with the microbial biofilm structure. **(A)** Anti-biofilm agents have limited activity against the biofilm community of microorganisms. **(B)** PLGA platforms release antibiotics in a sustained manner and boost the anti-biofilm agent’s site-specific delivery, as well as enhance their concentration in the microbial biofilm community. **(C)** PLGA increased the penetration of various anti-biofilm agents to the mucus and deeper layers of the biofilm. **(D)** PLGA has low cytotoxic effects on various human cells.

Based on the mentioned studies, PLGA NPFs could be considered an applicable approach for the local delivery of various antibiotics for the treatment of osteomyelitis. Since the bacterial biofilm makes this infection more resistant to the treatment, antibiotic-PLGA platforms could be used for the inhibition and destruction of the biofilm community, thereby facilitating the treatment of osteomyelitis. However, the reported data have been collected from *in vitro* evaluations, and for assessing the exact function of antibiotic-PLGA platforms in the treatment of osteomyelitis, this compound should be tested in further animal studies and clinical trials (**Figure 5**).

In addition to antibiotics, PLGAs have been used for the delivery of various natural compounds for the inhibition and destruction of the *S. aureus* biofilm community. Totarol, an ingredient of *Podocarpus totara*, is one of these natural compounds that in combination with PLGA was used for coating sutures (totarol/PLGA-coated sutures) to inhibit *S. aureus* growth and colonization. The results showed totarol/PLGA-coated sutures inhibited the growth of *S. aureus* (over a period of 15 days); additionally, this bacterium failed to form a biofilm on the coated sutures in comparison to uncoated sutures. In comparison to sutures coated only with totarol, sutures coated with PLGA and totarol had a better antibacterial activity. Thus, these findings suggested that totarol/PLGA-coated sutures have the potential to reduce the risk of postoperative biofilm formation on suture material and hence might decrease surgical site infection (Reinbold et al., 2017).

These data support the findings of Reinbold et al. who used the single-emulsion evaporation method and encapsulated totarol with PLGA microspheres. Totarol release was evaluated in a 90-day *in vitro* drug release assay, with the results showing a release of 53.76%. Notably, *S. aureus* could not form a biofilm on the surface of totarol-PLGA microspheres, while the biofilm of this bacterium was detected on unloaded PLGA microspheres. Furthermore, the authors did not detect a significant cytotoxic effect against human embryonic kidney cells. As such, these data suggested that the PLGA microspheres due to the great entrapment efficiency, low cytotoxicity, and slow release of totarol could be considered a new anti-biofilm agent that could be highly useful for the long-term therapy of bacterial infections (Reinbold et al., 2016).

In addition to totarol, in another examination, usnic acid (UA), a secondary metabolite of lichen was loaded on the PLGA-polyvinyl alcohol microspheres, after which matrix-assisted pulsed laser evaporation was performed to obtain thin coatings. UA-loaded microspheres not only efficiently suppressed *S. aureus* attachment, colonization, and biofilm formation for a period of up to 3 days under static conditions but also showed great biocompatibility (Grumezescu et al., 2014b). Hence, it seems that some drawbacks of natural compounds, such as low solubility in water that has been limited to their use in cosmetic formulations, topical ointments, and oral care, could be overcome by applying a PLGA-based drug delivery platform; nevertheless, confirmatory studies are required.

Nitric oxide (NO) has been considered a potential alternative for antimicrobial therapy due to the killing of bacteria in the planktonic and biofilm community. NO is an endogenously produced gas with considerable antimicrobial, vasorelaxation, and antitumor characteristics. Nonetheless, the clinical use of NO has been restricted because of some reasons such as instability during storage, short half-life, poor dose control, and lack of efficient localized and systemic delivery (Saraiva et al., 2011; Hasan et al., 2017). As a result, current research has employed various platforms to deliver therapeutic doses of NO in biological systems.

In this regard, a recently published study encapsulated NO precursor isosorbide mononitrate (ISMN) into the PLGA micro/nanoparticles (w/o/w emulsification/solvent evaporation technique). Both of the synthesized particles continuously released ISMN in physiological media over 3–5 days. Notably, ISMN-PLGA microparticles, with diameters of 3 µm and ISMN loading of 2.2% (w/w), were recognized as the best delivery system and indicated a remarkable anti-biofilm effect against *S. aureus* biofilms. The initial burst release followed by a continuous release of ISMN from PLGA microparticles boosted the diffusion of ISMN into the biofilm. Thus, the authors proposed that PLGA microparticles (but not NPs) have an acceptable potential to deliver sufficient levels of ISMN for inhibiting the *S. aureus* biofilm (Hasan et al., 2017).

In another investigation, the authors also prepared an NO-releasing film by fabricating doping dibutyhexyldiamine diazeniumdiolate (DBHD/N_2_O_2_) in PLGA. Next, this base layer was encapsulated with a silicone rubber top coating. PLGA in this compound acted as an NO release promoter and controller from the coating by providing protons through its hydrolysis products and intrinsic acid residues. DBHD/N_2_O_2_-doped PLGA-based NO-releasing coatings prevented the *S. aureus* and *E. coli* biofilm formation (over more than a week’s period). Accordingly, this NO-releasing platform could be considered as an anti-biofilm coating for the preparation of indwelling devices (Cai et al., 2012).

Finally, Hasan et al. prepared doped PLGA-polyethyleneimine/diazeniumdiolate NPs (PLGA-PEI/NO NPs) to enhance the NO penetration to the MRSA biofilm-infected wound. Notably, in the synthesized NPs, PLGA and polyethyleneimine/diazeniumdiolate were used as NP-forming polymer and NO donors, respectively. The synthesized NPs had an acceptable NO loading along with a prolonged NO release over 4 days. *In vitro* analysis revealed that PLGA-PEI/NO NPs were potently bound to the MRSA biofilm, leading to biofilm destruction and killing the planktonic bacteria upon biofilm dispersal. Additionally, the authors successfully formed MRSA biofilms on wound beds of diabetic mice and used the synthesized NPs for treating the wound infection. Treatment with PLGA-PEI/NO NPs removed the MRSA biofilm from the wound completely and accelerated wound healing in the non-diabetic and diabetic mice. Thus, this novel NO-releasing platform could be used for the treatment of biofilm-infected chronic wounds (Hasan et al., 2019).

## 5 Staphylococcus epidermidis

In addition to *S. aureus*, other types of coagulase-negative staphylococci such as *S. epidermidis* may also produce biofilms and be responsible for chronic wound maintenance. This bacterium is the most common microbe in device-associated infections and could colonize the surface of an implant and develop a biofilm (Rogers et al., 2009). In this regard, previous studies have used various approaches to suppress the adhesion and biofilm formation of *S. epidermidis*. PLGA NPFs are one of these approaches that have been used in recent studies for the delivery of antibiotics and natural compounds for the inhibition and destruction of the *S. epidermidis* biofilm, with this section reviewing these studies.

Niemelä et al. (2006) evaluated anti-biofilm characteristics of self-reinforced (SR) CIP-PLGA-releasing implant (the length of the implants was 30 mm and diameter was 3 mm) against *S. epidermidis*. SR-CIP/PLGA not only inhibited bacterial growth but also destructed the biofilm community by up to 99%. Notably, inhibition effects were not reported around implants without CIP (Niemelä et al., 2006b). These authors also compared this implant function with titanium in preventing *S. epidermidis* attachment plus biofilm formation in culture media. PLGA/CIP-releasing material significantly suppressed the *S. epidermidis* attachment and biofilm formation in comparison to the titanium and pure PLGA (Niemelä et al., 2006a). In another formulation, the authors also added CIP and bioactive glass to the PLGA 80/20 and polylactide (P (L/DL) LA 70/30; afterward, the mixture was extruded and self-reinforced. This compound, in line with the two mentioned CIP-release implants, also suppressed the bacterial attachment and biofilm formation (Ashammakhi et al., 2006). As a result, the scientists hypothesized that this CIP-containing biomaterial might play a role in the prevention and treatment of *S. epidermidis* biofilm-related infections such as trauma surgery and chronic osteomyelitis. Nonetheless, in the clinical infection, there may be several pathogenic microbes involved that may lead to the complicated interactions between the implant and the host; thus, *in vitro* findings should be confirmed by animal models and clinical trials.

In line with these results, in another examination, rifampicin- and calcium-eluting micropatterns were again used for the destruction of the *S. epidermidis* biofilm on orthopedic implant surfaces. Rifampicin-containing micropatterns consisted of a periodic array of 50-µm circular dots arranged 150 µm apart. For this purpose, PLGA and rifampicin were dissolved in an organic solvent with 100-nm biphasic calcium phosphate NPs suspended in the solution. Note that the rifampicin release rate was mainly influenced by the antibiotic loading in the micropattern, especially on the first day. The synthesized micropattern killed *S. epidermidis* before forming colonies, thus suppressing the biofilm community of this bacterium. Additionally, the presence of biphasic calcium phosphate in this nanostructure accelerated osteoblast cell differentiation without compromising cell proliferation (Gu et al., 2012).

These results support the findings of Chen et al. (2012) who deposed rifampicin-containing PLGA micropatterns onto the polycaprolactone/chitosan nanofiber meshes *via* ink-jet printing. This compound not only prevented the *S. epidermidis* biofilm formation but also boosted the osteogenic differentiation of preosteoblasts by upregulating the gene expression of bone markers. Hence, these data suggested that this PLGA-based nanostructure could be considered for covering orthopedic implant surfaces—thanks to proper anti-biofilm and healing effects.

A recently published study also used PLGA NPFs for the destruction of the *S. epidermidis* biofilm. In this regard, the emulsion solvent diffusion method was performed to synthesize clarithromycin-loaded + chitosan-modified PLGA (416.5 ± 4.6 nm size and 19.9 ± 1.7). CLSM analysis revealed that this compound showed high antibacterial and anti-biofilm effects against *S. epidermidis*. Microscopic study revealed that the produced NPs destroyed bacteria in the biofilm community’s deeper layer and eradicated the outer layer of the biofilm. Notably, the attachment and penetration capacity of NPs were enhanced by the modification of the NP surface with chitosan (Takahashi et al., 2015).

In addition to the mentioned antibiotics, Iannitelli et al. (2011a) used the solvent displacement method for the synthesis of carvacrol/PLGA NPs. Notably, carvacrol is an active compound of various natural products such as thyme and oregano. The synthesized NPs were 209.8 ± 7.2 nm in size and had -18.99 ± 3.01 zeta potential. Carvacrol/PLGA NPs showed good drug loading and encapsulation efficiency and were used for the destruction of *S. epidermidis*. The results revealed that these NPs would alter the properties of preformed *S. epidermidis* biofilms and significantly reduced the mechanical stability and elasticity of matured biofilms (Iannitelli et al., 2011a). Thus, as mentioned in the above studies, PLGA NPFs could lead to the acceptable penetration of anti-biofilm agents such as antibiotics and natural compounds to the deeper layer of the bacterial biofilm. However, more *in vitro* and animal model studies are required for better understanding of the PLGA-based platform interaction and *S. epidermidis* biofilm community.

Notably, other studies that used PLGA for the destruction of other microbial biofilm and bacterial biofilm-associated oral disorders are presented in **Tables 1** and **2**, respectively.

**Table 1:** Previous studies that used PLGA NPFs for inhibition of the microbial biofilm. PLGA: poly (lactide co-glycolide)

Year of publication(Reference)	Active Substance	NP Properties	Encapsulation Method	Microorganism	Outcomes
**2010** **(** Cheow et al., 2010a **)**	Fluoroquinolone antibiotics	170 ± 50 nm size	Emulsification-solvent-evaporation method	*E. coli*	Ciprofloxacin-loaded PLGA showed high drug encapsulation efficiency and anti-biofilm effect.
**2010** **(** Cheow et al., 2010b **)**	Levofloxacin	80 ± 30 nm size	Nanoprecipitation and emulsification-solvent-evaporation methods.	*E. coli*	Levofloxacin could be safely encapsulated into the PLGA without affecting its antibacterial activity.
**2011** **(** Said et al., 2011 **)**	Fusidic acid	200 nm to 2 µm	Electrospinning technique	*P. aeruginosa*, *S. aureus*	Eliminated planktonic bacteria and significantly suppressed biofilm community.
2015 **(** D’Angelo et al., 2015 **)**	Cationic antimicrobial peptides and colistin	300 nm size and +12.4 ± 2.1 mV ZP	Double-emulsification method	*P. aeruginosa*	NPs destroyed *P. aeruginosa* biofilm and showed a prolonged efficacy in biofilm elimination in comparison to the free colistin.
**2015** **(** Ma et al., 2015 **)**	Magnesium	NR	Low-temperature rapid prototyping technology	*S. aureus*,	Inhibited bacterial adhesion and biofilm formation.
**2016** **(** Kim et al., 2016 **)**	Clove oil and eugenol	NR	Various methods and Tanique	EHEC	This compound significantly inhibited biofilm formation.
**2017** **(** Takahashi et al., 2017 **)**	Silver	299.6 nm and −18.1 ZP	Emulsion solvent diffusion method	*Staphylococcus epidermidis*	Ag PLGA NPs could demonstrate high efficacy against biofilm infections.
2018 **(** Qiao et al., 2018 **)**	Terpyridine	80–100 nm size	Nanoprecipitation	*P. aeruginosa*	Terpyridine–micelle remarkably penetrated to the biofilm, suppressed biofilm development and reduced biofilm mass.
2019 **(** Iadnut et al., 2019 **)**	Propolis	500 nm size and ZP between -1.2 ± 1.1 mV and -3.9 ± 0.5 mV	Oil-in-water (o/w) single-emulsion solvent evaporation technique	*Candida albicans*	These particles suppressed invasion, hyphal germination, adhesion, and biofilm formation of *C. albicans*.
2019 **(** Simonetti et al., 2019 **)**	Pterostilbene and crude pomace extract	50–150 nm size	Microfluidic reactor with a flow-focusing configuration	*Candida albicans*	Synthesized NPs significantly inhibited biofilm formation in comparison to the free compounds.
2019 **(** Yang et al., 2019 **)**	AmB	287.8 ± 8.64 size and -10.9 ± 1.9 ZP	Double-emulsification method	*Candida albicans*	The combined used of 42 KHz ultrasound irradiation with AmB-NPs decreased biofilm biomass.
**2019** **(** Takahashi et al., 2019 **)**	Ionic liquids	300–700 nm size 55–60 mV ZP	Emulsion solvent diffusion method	*Staphylococcus epidermidis*	Showed antibacterial activity against bacteria in the biofilm community.
**2020** **(** Deepika et al., 2020 **)**	Rutin and benzamide	285 ± 6 nm size, **-**17.6 ± 0.6 ZP	Oil-in-water emulsion solvent evaporation technique	*P. aeruginosa*, *S. aureus*	The synthesized NPs disrupted the membrane and biofilm surface.
**2020** **(** Liu et al., 2020 **)**	Platensimycin	175.6 ± 2.6 nm to 218.1 ± 2.7 nm size, +17.2 mV ZP	Emulsification solvent evaporation method	*S. aureus*, *S. epidermidis*	Compared to free compound, the synthesized NPs killed *S. aureus* in a macrophage cell infection model and inhibited the biofilm formation.
**2020** **(** Mao et al., 2018 **)**	Silver and Hydroxyapatite NPs	1,986.8 nm (Hydroxyapatite) and 469.3 nm (silver NPs)	Solid-in-oil Nano suspension method	*S. aureus* *MRSA*	Effectively increased osteogenesis of MC3T3-E1 cells, reduced bacterial adhesion and biofilm formation.
**2020** **(** Permana et al., 2020 **)**	Dissolving microneedles of doxycycline	207–247 nm size, -6.3 to 29.5 ZP	A double-emulsion (water-in-oil-in-water) solvent evaporation method	*P. aeruginosa*, *S. aureus*	The use of nano carrier enhanced anti-biofilm properties and dermatokinetic profiles of doxycycline.
**2020** **(** Zodrow et al., 2012 **)**	CIN and CAR	CIN-PLGA 519 ± 35 nm size, CAR-PLGA nm 502 ± 13	Various methods and Tanique	*P. aeruginosa*, *S. aureus* *E. coli*	CIN-PLGA impaired *S. aureus* biofilm development. CAR-PLGA decreased the biofilm community of *S. aureus* and *E. coli*.
**2020** **(** Ma et al., 2020 **)**	Magnesium	NR	Low-temperature rapid-prototyping technique	*Staphylococcus epidermidis*	This compound inhibited the bacterial growth and biofilm formation.
2021 **(** Gumus et al., 2020 **)**	Juglone	207.60 ± 1.99 nm size and -25.7 ± 1.2 mV ZP	Single-emulsion solvent evaporation method	*Candida albicans*	These particles significantly suppressed the formation of the biofilm and preestablished biofilms in comparison to the fluconazole and free Juglone.
**2021** **(** Tan et al., 2021 **)**	Ampicillin	194 nm size and 20.9 mv ZP	Combination approach of emulsion solvent evaporation and lipid thin-film rehydration	*Enterococcus faecalis*	This compound increased the efficacy of ampicillin against the planktonic and biofilm community of bacteria.

NPs, nanoparticles; AmB, amphotericin B; ZP, zeta potential; NR, not reported; MRSA, methicillin-resistant Staphylococcus aureus; CIN, cinnamaldehyde; CAR, carvacrol; PLGA, poly (lactide co-glycolide).

**Table 2:** Different PLGA NPFs that have been used for inhibition of bacterial biofilm-associated oral disorders.

Year of publication(Reference)	Active Substance	Drug-platform	Encapsulation Method	Microorganism	Outcomes
**2020** **(** Akram et al., 2021 **)**	Chlorhexidine	Chlorhexidine-loaded mesoporous silica NPs modified with PLGA	Sol-gel technique	*Streptococcus mutans*	The modified NPs with PLGA showed more profound anti-biofilm properties against *S. mutans.*
**2019** **(** Mahmoud et al., 2019a **)**	BAR (SspB Adherence Region)	10:90 PLGA/polyethylene oxide Polymeric electrospun fibers.	3	*Porphyromonas gingivalis, Streptococcus gordonii*	This formulation suppressed the biofilm formation and disrupted the established dual-species biofilms.
**2020** **(** Arafa et al., 2020 **)**	CIP	CIP-PLGA NPs, CIP-PLGA NPs coated with chitosan	Double-emulsion solvent evaporation technique	*Enterococcus faecalis*	CIP-PLGA NPs coated with chitosan showed the best antibacterial and anti-biofilm effect in comparison to the CIP-PLGA NPs and CIP solution.
**2020** **(** Sebelemetja et al., 2020 **)**	DVA	DVA-PLGA/PEG polymeric NPs	Double-emulsion (w/ o/w) method	*Streptococcus mutans*	Decreased acid production and suppressed biofilm formation by 92%.
**2018** **(** Fan et al., 2018 **)**	Ag+ and Ca2+	AgCa-PLGA submicron particles	Modified water/oil/water (w/o/w) emulsification solvent evaporation method.	*Enterococcus faecalis*, *Porphyromonas gingivalis*	The synthesized NPs (by ultrasonic activation) suppressed the colonization of *P. gingivalis* and *E. faecalis* on dentin.
**2018** **(** Mahmoud et al., 2018 **)**	BAR (SspB Adherence Region)	BAR-encapsulated PLGA and mPEG-PLGA NPs	Double-emulsion technique	*Porphyromonas gingivalis, Streptococcus gordonii*	These NPs effectively reduced the biofilm formation and eliminated the preformed biofilm.
**2017** **(** Li et al., 2017 **)**	AMP (KSL-W)	KSL-W-loaded PLGA/chitosan composite microspheres	Electrospraying and combined crosslinking-emulsion methods	*Fusobacterium nucleatum*	These NPs showed prolonged antibacterial and inhibitory effects.
**2017** **(** Kalia et al., 2017 **)**	BAR (SspB Adherence Region)	BAR-modified PLGA NPs	Oil-in-water (o/w) single-emulsion technique	*Porphyromonas gingivalis*, *Streptococcus gordonii*	These NPs more effectively suppressed the *P. gingivalis/S. gordonii* adherence and biofilm formation in comparison to the equimolar amount of free peptide.
**2017** **(** Freire et al., 2017 **)**	Antiserum against DNABII family (anti-DNABII)	Anti-DNABII/PLGA microsphere	Modified double-emulsion technique	*Aggregatibacter actinomycetemcomitans*	These NPs increased the immune system ability to eliminate the bacterial biofilm.
**2021** **(** Geremias et al., 2021 **)**	Tea tree oil and furan-2(5H)-one.	PLGA electrospun membranes incorporated with tea tree oil and furan-2(5H)-one	Electrospraying	*Streptococcus mutans*	Results indicated a remarkable decrease in bacterial adherence on the synthesized membranes.
**2019** **(** Mahmoud et al., 2019b **)**	Peptide (BAR) derived from *Streptococcus gordonii*	Peptide-Modified PLGA NPs	Single-emulsion technique	*Porphyromonas gingivalis*	These NPs inhibited the *P. gingivalis* colonization, biofilm formation, and bacterial virulence in a mouse model of periodontitis.

NPs, nanoparticles; CIP, ciprofloxacin; DVA, Dodonaea viscosa var. angustifolia; PEG, poly ethylene glycol; mPEG, methoxy-polyethylene glycol; AMP, antimicrobial peptide; PLGA, poly (lactide co-glycolide).

### 5.1 Conclusion With Future Recommendations for Poly Lactic-*Co*-Glycolic Acid Therapy

Bacterial infections caused by MDR pathogens can put public health and human lives at risk. Additionally, due to the low penetration of antibiotics, biofilm community of microorganisms extend antibiotics resistance. In this case, nanotechnology and anti-biofilm agents could be used together to fight MDR pathogen biofilms. Today, PLGA NPFs are the basis for medical applications in various fields used by chemists, biologists, and physicians, as well as academic and industrial groups. In the future, we expect new design techniques to further expand the use of PLGA and create efficient delivery systems, which will play an important role in the inhibition and destruction of the microbial biofilm, especially in the MDR biofilm community. For instance, it could be possible to enhance the efficacy of PLGA NPFs by modifying particle surfaces with a cationic polymer such as chitosan. This procedure potentially enhances the interaction of PLGA NPs with anionic mucopolysaccharide in the biofilm and maximizes biofilm exposure to various anti-biofilm agents. Notably, the relative nontoxic nature of PLGA degradation products and its degradability under physiological conditions make it an applicable candidate for creating tissue-engineering scaffolds. However, some PLGA disadvantages such as suboptimal bioactivity and low hydrophilicity limit its applications in a clinical setting and should be considered in future investigations.

This review shows that PLGA could be used for the delivery of various anti-biofilm agents such as antibiotics, natural compounds, NO, and enzymes. However, the data about exact interactions of PLGA NPFs and biofilm structure of microorganisms, product composition, manufacturing procedure for NPs, final particle size, and route of administration, as well as drug release rate requirements are limited. Furthermore, there are no proper standards and guidelines for testing the efficacy, safety, and performance of PLGA NPFs against the microbial biofilm. Thus, more investigations are needed to evaluate the mechanisms by which PLGA NPs suppressed the biofilm formation and eliminated the mature biofilm.

## Author Contributions

AS and SH conceived and designed the study. AS, ZC, and SH contributed to the comprehensive research and wrote the paper. EG-R and ENZ participated in article editing. Notably, all authors have read and approved the article.

## Conflict of Interest

The authors declare that the research was conducted in the absence of any commercial or financial relationships that could be construed as a potential conflict of interest.

## Publisher’s Note

All claims expressed in this article are solely those of the authors and do not necessarily represent those of their affiliated organizations, or those of the publisher, the editors and the reviewers. Any product that may be evaluated in this article, or claim that may be made by its manufacturer, is not guaranteed or endorsed by the publisher.

## Abbreviations

EPS, extracellular matrix; NPs, nanoparticles; PEG, polyethylene glycol; PLGA, poly (lactide co-glycolide); CIP, ciprofloxacin; SEM, scanning electron microscope; W/O/W, water-in-oil-in-water; OMF, oscillating magnetic field; CLSM, confocal laser scanning microscopy; OSA-HA, octenyl succinic anhydride-modified low-molecular weight hyaluronic acid; MBG, mesoporous bioactive glass; PVA, polyvinyl alcohol; UA, usnic acid; NO, nitric oxide; ISMN, isosorbide mononitrate; DBHD, dibutyhexyldiamine diazeniumdiolate; MRSA, methicillin-resistant *S. aureus*; MDR, multidrug resistant.
